# The role of gender in the spread of HIV and AIDS among farmworkers in South Africa

**DOI:** 10.4102/phcfm.v10i1.1668

**Published:** 2018-11-08

**Authors:** Ntombifikile E. Klaas, Gloria Thupayagale-Tshweneagae, Thuledi P. Makua

**Affiliations:** 1 Department of Health Studies, University of South Africa, South Africa

## Abstract

**Background:**

Gender inequality and men’s perceived sexual and economic superiority over women are central to human immunodeficiency virus (HIV) infection. The farming community in which the participants in the study live operates along such patriarchal lines, with the men making the important decisions for their families.

**Aim:**

To explore and describe the role of gender in the spread of HIV and acquired immunodeficiency syndrome (AIDS) among farmworkers in South Africa.

**Setting:**

The study was conducted in the Levubu farms, Vhembe district, Limpopo Province, South Africa.

**Methods:**

The researchers adopted a qualitative, explorative and descriptive research design with in-depth semi-structured interviews. Purposive and convenience sampling methods were used to select participants who met the inclusion criteria. Data collected were thematically analysed using Creswell’s data analysis method. Lincoln and Guba’s model to ensure trustworthiness and ethical standards were applied.

**Results:**

The findings of the study clearly indicated that powerlessness and lack of decision-making by female farmworkers was common as female farmworkers were dependent on their male partners to make decisions in the workplace as well as decisions regarding sexual matters in a relationship.

**Conclusion:**

The main conclusion drawn from the findings were that farmworkers are continuously exposed to exploitation and disempowerment in a variety of ways with very little support from their supervisors, which makes them vulnerable to contracting HIV.

## Introduction

Globally, an estimated 36.7 million people were living with HIV in 2016; 34.5 million of the 36.7 million were adults, 17.8 million were women above the age of 15 years, 1.8 million were newly infected with HIV and 1 million people died because of AIDS-related illnesses. In June 2017, 20.9 million people were accessing antiretroviral treatment (ART). Sub-Saharan Africa is home to more than half of people living with HIV globally. South Africa has the biggest HIV epidemic profile in the world, with an estimated 7.1 million people living with HIV in 2016. South Africa accounts for a third of all new HIV infections in sub-Saharan Africa. In 2016, there were 270 000 new HIV infections and about two-thirds were women. Hundred and ten thousand South Africans died from AIDS-related illnesses. In June 2016, 3.7 million people were accessing ART. South Africa has the largest ART programme in the world and these efforts have been largely financed from domestic resources.^1^

The human immunodeficiency virus (HIV) is the leading cause of death and disease in women of reproductive age. In low- and middle-income countries worldwide, the prevalence among young women aged 15–24 years is on average three times higher than among men of the same age.^2^ In sub-Saharan Africa, 60% of people living with HIV are women. This perspective is shared by Wathula (2016), among others, that over half (58%) of those living with HIV in sub-Saharan Africa are women, who in general acquire HIV infection at least 5–7 years earlier than men.^3^

The South African National AIDS Council (SANAC) reported the HIV prevalence among young women in South Africa to be nearly four times greater than that of men of the same age. In 2016, young women between the ages of 15 and 24 made up 37% of new infections in South Africa. To try and reduce this high rate of infection, young women and adolescent girls who are considered at high risk of HIV infection are now being offered pre-exposure prophylaxis.^4^ According to the United States (US) Agency for International Development, poverty, the low status of women and gender-based violence (GBV) have all been cited as reasons for the disparity in HIV prevalence between genders. Indeed GBV contributed to an estimated 20% to 25% of new HIV infections in young women.^2,3^ Gender inequalities and men’s perceived sexual and economic superiority over women can have significant health implications. These perceptions are central to HIV infection.^5^

Gender issues impact on all aspects of society and culture, from economic growth to the health and well-being of the overall population. Despite the importance of gender equity, discrimination against women is pervasive. The destructive effects can be seen in lower global literacy skills in women, high regional infant and maternal mortality rates, poor representation in government at local and national level and the high percentage of violence against girls and women worldwide. Researchers and policymakers have long recognised that gender plays a role in the vulnerability to HIV and/or AIDS and its impacts in every region in the world.^4,6^

According to the feminisation of poverty theory, women’s power inequalities render them especially vulnerable to HIV infection.^3^ In a patriarchal culture, men are seen as dominant both in the family as well as in society at large. In addition, men are seen as more intelligent than and superior to women. As a result of their societal roles, women and girls face a number of unique challenges that affect their ability to protect themselves from HIV and/or AIDS and its overwhelming effects. This is evidenced by the disproportionate impact of the epidemic on women, especially in sub-Saharan Africa.^7^

In every society, there are differences in the distinct behaviour patterns of men and women to qualify them as belonging to two different cultures and/or subcultures. In patriarchal male-dominated societies, women’s subordination and male dominance are so pronounced that their subcultures are literally separated by a world of difference. One such society is South Africa.^8^

Women subordination is rooted in a set of customary and legal constraints that block women’s entrance to the public world. Because society holds a false belief that women are naturally less intellectual and physically less capable than men, it tends to discriminate against women in the academics, forum and marketplace. The farming community in which the participants in the study live operates along such patriarchal lines, with the men making the important decisions for their families.^8,9^

The prevalence of HIV is very high among farmworkers, with almost four people out of every ten being HIV-positive. This is stated to be the highest prevalence ever published in southern Africa among a working population. Congruent to these findings, a study in Zimbabwe revealed that 42% of the 380 farmworkers were HIV-positive. Several studies also noted high rates of HIV prevalence (35%) among farmworkers and mineworkers in Zimbabwe. Despite the high HIV prevalence, only a marginal proportion of HIV-positive farm- workers were on ART.^8,9,10^

Female farmworkers are vulnerable to HIV and/or AIDS, similar to female migrant workers in the mining and agricultural sectors in Africa, where ‘feminisation’ of AIDS is most visible. South African farmworkers are the most vulnerable members of the South African work force, earning the lowest wages, with women earning even less than men. Commercial farms in Southern Africa, particularly in the Mpumalanga, Limpopo and Free State provinces, employ a significant number of migrant workers, including those from other African countries, mainly Mozambique, Zimbabwe and Lesotho.^8,9^

Seasonal farmworkers are a common feature on most South African farms and this increases the possibility of female workers contracting HIV. The majority of those who work seasonally are female farmworkers in very poor living conditions. Desperate to secure employment for the duration of the harvest, young women have sex with male supervisors, known as ‘indunas,’ in exchange for a job. Becoming the girlfriend of a supervisor for a season guarantees accommodation on the farm and better working conditions. Poor living and working conditions, separation from families, physically demanding work with low wages, limited access to and inadequate healthcare services predispose farmworkers to HIV and/or AIDS.^3,8^

### Objective

This study aimed to determine the extent of gender roles in contributing to the spread of HIV among farmworkers. This was done by exploring and describing the perceptions of the farmworkers related to the role of gender in the spread of HIV and/or AIDS among farmworkers in South Africa.

## Research methodology

### Research design

A qualitative approach using exploratory and descriptive designs were used in order to explore and describe the role of gender in the spread of HIV and/or AIDS among farmworkers in Tshitwani and Barota farms in Vhembe district, Limpopo province, South Africa. The researcher used liberal feminism as a framework to explore the role of gender in the spread of HIV among farmworkers. Liberal feminism argues that women have been discriminated against by virtue of their sex throughout the world.^7^ The choice of this research location was informed primarily by high levels of HIV prevalence and poverty in this area.

The study conducted by Colvin on HIV infection among farmworkers in Malelane, Tzaneen and Musina showed that 39.5% of 2798 workers on the 23 Limpopo farms where HIV programmes were already implemented were HIV-positive. The 39.5% infection rate on these farms is twice the national UNAIDS prevalence of 18.9% in South Africa. This is also the highest prevalence ever reported in any working population.^8,9^

### Study setting and participants

Purposive and convenience sampling were used to choose the participants from the Levubu farms, Vhembe district, Limpopo province who met the inclusion criteria and were willing to participate.^11^

The choice of this research location was informed primarily by high HIV prevalence and the fact that the farmworkers have limited access to healthcare services, which is why their healthcare needs are serviced by a mobile clinic. The HIV prevalence on the Levubu farms is twice the UNAIDS national prevalence percentage of 18.9% in South Africa.^8,9^ The situation described above made the research setting well suited to investigate. The researcher interviewed 17 farmworkers, 3 supervisors, 1 professional nurse and an ex-human resources (ex-HR) manager from Tshakhuma farm. No farm managers or owners participated in the study.

### Data collection

Data were collected from 22 participants through in-depth semi-structured interviews supported by recordings and field notes. Participants were diverse in terms of age, gender, ethnicity, education and occupation. Fifteen female and seven male participants were interviewed. Written consent was obtained from the participants prior to data collection. Semi-structured interviews were conducted at the farm manager’s office, under the tree and even in the field where the farmworkers were working. As difficult as it was to control the noise levels, the researcher had a chance to observe the participants in their natural setting. Interviews were conducted in each participant’s preferred language (Tshivenda, Xitsonga and isiZulu).

Both the researcher and the research assistant altered roles in conducting the interviews and taking field notes. This was determined by the preferred language of the participants. The Tshivenda and Xitsonga interviews were conducted by the research assistant, as he was fluent in these languages, and the researcher conducted interviews in isiZulu. All interviews were initiated from a broad central question: ‘What is the role of gender in the spread of HIV among farmworkers?’ Various communication skills like probing, paraphrasing and reflecting were used by the researcher and the research assistant during the interviews. Each interview lasted for approximately 30 min. The researcher and research assistant also used naturalistic observation as a data collection method. Data were collected until saturation was reached and no new information was obtained. The pilot study was carried out prior to data collection and the necessary adjustments were made to the study.^12^

### Data analysis

The researcher analysed the data by using Tesch’s analysis method. The recorded interviews were transcribed verbatim. The researcher picked the most interesting and brief interview and jotted down any ideas that came to mind. After completion of several participants’ responses, a list was made of all similar topics that emerged. The identified topics were then abbreviated into codes, the most descriptive wording for similar topics was identified and categories were formed. When the researcher reread the transcripts to commence with the analysis, she picked up that some transcripts lacked a comprehensive description. The researcher liaised with the relevant participants to arrange follow-up interviews. Categories, subcategories and themes were identified through clustering of descriptive phrases from transcriptions. A final decision on the abbreviation of each category was made and the data belonging to each category was assembled. Finally, preliminary analysis was done by the researcher. In order to avoid bias, the transcriptions together with field notes were sent to an independent coder for validation.^12,13^

### Trustworthiness

Guba’s model of trustworthiness was utilised in order to ensure validity and reliability within the study. The strategies of credibility, conformability, dependability and transferability were implemented. Credibility was ensured by using prolonged and varied field experience, interviewing process, peer review, reflexivity and triangulation. The credibility of the findings was ensured through prolonged and varied field experience, triangulation, member checking and peer examination. Conformability was ensured by triangulation and using bracketing to avoid contamination of data.^12^

Richness of the data was preserved by using quotations. Dependability was ensured by taking field notes and observing non-verbal cues throughout the interview, and an independent coder assisted the researcher in analysing and interpreting the data collected. Transferability was further ensured by using various data collection methods like interviews, field notes and tape recording.^13,14^

### Ethical consideration

Ethical clearance was obtained from the Research and Ethics Committee of the Department of Health Studies at the University of South Africa (UNISA) prior to data collection (reference number HSHDC/443/2015). Institutional consent was also granted by the Tshakhuma and Tshitwani farms prior to data collection. Informed consent was obtained from the participants before the interviews; a written consent form was handed out and the purpose of the study was explained. Specifically, the participants were informed in writing about the objectives of the study and its benefits in the prevention of HIV and AIDS. The right of patients to refuse participation without fear of intimidation or victimisation was also described before they were requested to sign the form for data collection. The ethical principles of respect for persons, beneficence and justice were upheld.

## Results

The researcher used liberal feminism as a framework to explore the role of gender in the spread of HIV among farmworkers.^7^ The study participants raised many issues in relation to their perceptions regarding this. The participants were described according to their demographic characteristics (age, gender, employment status, job title, duration of employment and employment status) (see Table 1).

**TABLE 1:** Demographic characteristics of the participants.

Participant number	Gender	Age (years)	Job title	Employment status	Duration employed (years)
1	Female	36	Farmworker	Permanent	7
2	Male	26	Farmworker	Part-time	2
3	Male	48	Supervisor	Permanent	10
4	Female	53	Farmworker	Permanent	11
5	Female	44	Farmworker	Permanent	6
6	Female	39	Farmworker	Permanent	7
7	Male	32	Farmworker	Permanent	6
8	Female	48	Farmworker	Permanent	10
9	Female	51	Farmworker	Permanent	10
10	Male	40	Farmworker	Permanent	7
11	Male	49	Supervisor	Permanent	6
12	Male	49	Farmworker	Part-time	6
13	Female	47	Farmworker	Permanent	7
14	Female	51	Farmworker	Permanent	12
15	Female	42	Supervisor	Permanent	9
16	Female	59	Farmworker	Permanent	26
17	Female	51	Farmworker	Permanent	12
18	Female	48	Farmworker	Permanent	10
19	Female	49	Farmworker	Permanent	11
20	Male	32	Farmworker	Part-time	3
21	Female	31	Ex-HR manager	Unemployed	10
22	Female	36	Registered Nurse	Permanent	7

HR, human resources.

The 22 participants included in this study comprised 7 men and 15 women. Even though one of the limitations of this study was a smaller sample size, the information of the participants in this study verified that the majority of farmworkers were women. Three participants – one woman and two men – were supervisors. Despite the fact that the majority of the participants in this study were women, very few were in a management position. This finding is consistent with other studies which revealed that there is an unequal power distribution among men and women who are farmworkers. It was also evident that supervisors and managers use their position to exploit the women. This information is confirmed by the participants at a later stage and it is consistent with previous research findings that gender division is one of the most significant inequalities which cuts across all social and income groups.^9,10^

According to Statistics South Africa, the Vhembe district settlement pattern is largely rural, with the majority of the population being women under the age of 20 years.^8,15^

The age of the participants ranged between 26 and 59 years. Participant 7 and 11 were mechanics, participant 21 was an ex-HR manager for Tshakhuma farm and participant 22 was a healthcare professional from Tshakhuma clinic.

The themes related to the challenges experienced by farmworkers who were interviewed are presented below.

### Gender inequality increasing the spread of HIV

The first theme which emerged during data analysis was gender inequality increasing the spread of HIV. Participants highlighted that the women are ‘powerless’ and gender inequality was still common in the farming community.

The following statements bear evidence:

‘There are two female supervisors and six male supervisors.’ (Participant 3, male, 48 years)

‘There is no 50/50 here and it won’t work in the farm. Will women be able to lift up crates and climb trees? We need to be realistic.’ (Participant 11, male, 49 years)

‘We must stop forcing matters. Women come second; men are the head of the household. (*Mufumakadzi uda nga murau, munna ngi thoho ya mudi.*)’ (Participant 12, male, 49 years)

‘Men use their position. (*Vhanna vha shumisa tshimo tshabo.*)’ (Participant 12, male, 49 years)

‘It used to happen before when we used to get our salaries in envelopes. The managers will give more money to their girlfriends. It is better now because our salaries are deposited in our bank accounts.’ (Participant 14, female, 51 years)

‘Women want to date foremans and supervisors because they will give them money. Women say if you need money, you must go to men. (*Abafazi bafuna amaforomani nama manager ngoba akunamali. Abafazi bathi imali itholakala emadodeni.*)’ (Participant 14, female, 51 years)

‘My husband forces me into having unprotected sex because he says I am his wife and that he paid lobola for me. (*Indoda yami iya ocansini name ngenkani ingafakanga ikhondomu ngoba ithi ngingu nkosikazi wayo futhi ikhokhe ilobolo*.)’ (Participant 15, female, 42 years)

The majority of participants expressed that women end up in a relationship with a manager or supervisor because they want to work less, have more money and secure their employment. Some women expressed that they feel victimised and forced to be in such relationships.^3,8,10^

Women are generally not socialised to initiate sexual activity. This task is normally considered to be part of a man’s role.^16^ Men perceive themselves to be naturally superior to women and often consider it a cultural right to have multiple partners. Such behaviour is generally equated with notions of normative masculinity. Finally, women are commonly implicated for bringing HIV into a relationship while their male counterparts are culturally absolved of blame for the disease.^17^

### Socio-economic factors linked to HIV and AIDS

Socio-economic factors linked to HIV and/ or AIDS emerged as the second theme during data analysis. Socio-economic vulnerability was identified as a category under this theme and poverty and low income as well as illiteracy were the subcategories.

#### Poverty and low income

Participants in this study could relate to the challenges presented by poverty and low income among farmworkers.

‘Money is too little to cover the expenses. The company is not having enough money to pay the workers. The working hours have been cut from nine to seven hours and salaries are now less. The working conditions seem to be a challenge. Most women are single, so they end up having relationships with supervisors and managers to have more money.’ (Participant 3, male, 48 years)

‘The government must increase the salaries of the farmworkers.’ (Participant 14, female, 51 years)

‘There is no money in the farms. Women want to date foremen and supervisors because they will give them money. Women say if you need money, you must go to men, men love us but love is like fire, you die in the name of love. (*Emapulazini akuna mali. Abafazi bafuna amaforomani nama manager ngoba akunamali. Abafazi bathi imali itholakala emadodeni. Amadoda ayasithanda, kodwa uthando lufana nomlilo, luyashisa. Kuyafiwa othandweni*.)’ (Participant 14, female, 51 years)

‘If you have two boyfriends each one will give you R100, it is better than nothing.’ (Participant 8, female, 48 years)

‘Women want money. (*Vhafumakadzi vha funa masheleni.*)’ (Participant 11, male, 49 years)

‘HIV is spreading fast because it’s competition, everyone wants to date the same person.’ (Participant 2, male, 26 years)

‘I am poor, my parents are poor, the farmworkers are poor, and it is from generation to generation.’ (Participant 3, male, 48 years)

Local farmworkers are poor and worse off than many other groups. Again poverty, especially within a context of oppression and exploitation, could lead to violence. Women farmworkers are extremely vulnerable, as they are discriminated against, in terms of access to employment and lower wages, and are completely dependent on the men for housing.

#### Illiteracy

Participant 3 and 10 were quite vocal about the high levels of illiteracy among farmworkers. They linked the illiteracy levels to the spread of HIV among farmworkers.

The following narrative statements support the above information:

‘HIV spreads rapidly on the farms because people are illiterate. The information on HIV is available in a form of posters which are in English. So how are farmworkers expected to read these posters? Condoms are used by people who can read because they understand the importance of using them. Put them there (condoms), nobody will take them. Some people still don’t believe that HIV exists because they ask where was HIV all along?’ (Participant 3, male, 48 years)

‘Farmworkers are not educated.’ (Participant 4, female, 53 years)

‘People don’t want to use them (condoms), they are not interested. People who use condoms are those who understand and can read. Put them there, nobody will take them.’ (Participant 3, male, 48 years)

The relationship between HIV and/or AIDS is complex and dynamic. Research shows that women continue to have less knowledge about prevention of HIV transmission as compared to their male counterparts. Women have a significantly lower rate of multiple partners. These findings are consistent with lower rates of condom use at last sex, which suggests that women may not have equal power to negotiate safe sex even if they have the same knowledge about prevention as men.^1^

## Discussion

The purpose of this study was to explore and describe the role of gender in the spread of HIV and/or AIDS.

As much as HIV-positive women may want to practice safer sex in order to avoid reinfection, the reality is that they may not be in a position to do so. Gender roles of women and, in particular, the need to maintain connections in relationships at the cost of one’s own health are key issues for all women living with chronic diseases, especially those who are seropositive.^17,18^ Women living with HIV will continue to be sexually active with men, silencing their voices during times of sexual intimacy in order to maintain a connection with their partner, rather than taking care of themselves. With direct requests, these women will not protect themselves against further strains of HIV and other sexually transmitted diseases (STDs) and they will continue to infect others and weaken the already suppressed immune system.^19^

The gendering of boys to reinforce their masculinity and cultural practices which condition women to be submissive increase the risk of HIV infection. Men’s disproportionate power over women plays a critical role in the spread of HIV by subordinating women and rendering them powerless in negotiating safer sex practices.^18,19^

The expectations for sexual passivity in women, along with the priority given to male sexual pleasure, also make it difficult for women to be an equal partner in deciding the terms of sexual activity, including negotiating safer sex practices. The power imbalance between men and women also translates into economic dependency for women. In most societies, men have greater control and access to productive resources. Women may feel pressured to stay in risky or abusive relationships with men because of the economic consequences of leaving such relationships. Limited income-earning opportunities are a common challenge for girls and women around the world. Women may be forced to exchange sexual favours for money or gifts in order to meet their basic needs, support their families, pay for school, or even to enhance their social power.^8,9^

The findings regarding poverty and low income seemed powerful enough to compel the women farmworkers to engage in multiple relationships to make ends meet. Poverty can also make it difficult for people infected with HIV to concern themselves with long-term risks. They may believe that their lives will be short because of poverty; thus, they have nothing to lose by risking infection in the quest for survival. However, research has shown that in the context of poverty, individuals are likely to engage in risky sexual behaviours as an attempt to remedy their situation. For instance, poor individuals are likely to make a decision around the constant assurance of human resources and basic needs such as food, shelter, etc., and not necessarily around HIV and/or AIDS prevention. This illustrates that poverty exposes individuals to situations that make them vulnerable to HIV.^1,3,8,9^

According to Statistics South Africa, 57% of the provincial population was living in poverty in 2016. Limpopo province is one of the poorest, rural provinces of the nine provinces in South Africa.^15^

The ability to use condoms and negotiate safe sex is deeply embedded in power relations that cannot be separated from financial independence. Even when individuals know about HIV and how it is transmitted, it is possible that they may not practice what they know because of poverty and food insecurity.^1^ Furthermore, evidence suggests that the poor and less educated are more likely not to use condoms compared to economically advantaged people. Possible explanations advanced include economic dependence on partners. A study in Cape Town also found that young men in households with 10% higher poverty rates were less likely to report condom use at last sex. Education and a high economic status is said to be associated with increased condom use and reduction in multiple partners.^17,19^

The expectations for sexual passivity in women, along with the priority given to male sexual pleasure, also makes it difficult for women to be an equal partner in deciding the terms of sexual activity, including negotiating safer sex practices. The power imbalance between men and women also translates into economic dependency for women. In most societies, men have greater control and access to productive resources. Women may feel pressured to stay in risky or abusive relationships with men because of the economic consequences of leaving.

HIV and/or AIDS remain a major problem despite 30 years of information sessions and condom distribution. It is, therefore, important that HIV programmes be reviewed and customised. Each programme should be made accessible and promoted to a specific target group. The researcher is of the opinion that HIV awareness programmes should be designed in such a way that they ensure relevance to their targeted communities’ realities. These programmes should impact on the attitudes, beliefs and behaviours to change risky social norms, practices and misconceptions.^16,17^

Research shows that women continue to have less knowledge about prevention of HIV transmission compared to their male counterparts. Farmworkers in this study universally reported that they had heard of HIV and/or AIDS and attested that information is available in the form of posters. Lack of comprehensive information about HIV and/or AIDS and the fact that the posters are written in English have negative implications for those infected and affected, as it leads to social stigma and discrimination.^19,20^

Globally, HIV is the major cause of mortality among women 15–49 years of age, with HIV-related deaths being 30% higher in African women than African men. Worldwide, women continue to have less knowledge about prevention and more misconceptions about routes of HIV transmission than men.^1,5,16^

## Limitations

The qualitative nature of the study limited the possibility of generalising the research results to all other farmworkers in South Africa or even other countries. The sample of the study comprised only 22 participants aged between 26 to 59 years who met the inclusion criteria. This small size limits generalisation and external validity of the findings. This in turn limits the scope of the research as it is difficult to assess the impact the research has on the real world. The interviews were conducted in indigenous languages which included Tshivenda, Xitsonga and IsiZulu and translations of the interviews were time consuming.

## Recommendations

It is recommended that the farm owners, farmworkers and healthcare workers should work together in order to curb the spread of HIV. Gender-sensitive strategies should be developed and adhered to by the relevant stakeholders. Oppressive and only men-favouring cultural beliefs and practices should be amended to address the needs of women. HIV vulnerability programmes need to cater for different age groups, different literacy levels and both sexes. Involvement of men in gender equity programmes is crucial. These programmes should practice justice for all and treat everyone equally irrespective of age, culture, socio-economic status and gender. Breaking down sexual behaviour stereotypes can pave the way for more equitable and mutually supportive relationships that are conducive to behaviour change. This would set the stage for both women and men to be active agents of transformation in a collaborative effort leading to sound HIV prevention outcomes. The farming community should be taught to have respect for and a positive attitude towards women’s roles in day-to-day practices in the workplace and society.

Because there is still poor representation at local and national level, the farm owners and supervisors should be challenged by gender equity programmes as well as the labour department to allocate jobs fairly according to qualifications and experience and avoid discriminating against women.

Women should benefit from affirmative action in the employment process because of their historical disadvantage and current level of lower achievements when compared to men.

Farmworkers should understand the role of education and use all efforts to improve their educational achievements. The government can play a crucial role by liaising with the farm owners and building more schools which are accessible to the farming community. Initiatives aimed at continuing to raise awareness of HIV and/or AIDS on the farms should be culture sensitive and be at the level of the farmworkers in order to facilitate prevention of HIV.

## Conclusion

In order to address the needs of farmworkers, it is important to provide health promotion which is gender-sensitive to avoid exacerbating the existing gender inequality among farmworkers. Gender inequality and disempowerment of women are expressed as factors increasing the HIV vulnerability of female farmworkers as they find themselves in a compromised situation of being discriminated against by their male counterparts, their families and their employers. The researcher believes that it is crucial that men be brought into the mainstream of HIV and/or AIDS policies and programmes to challenge their social constructions and address their psycho-social and socio-economic vulnerabilities.

Gender equality and social change will also encourage men to become important partners in curbing the spread of HIV. This is particularly relevant to farmworkers in South Africa, where the highest rates of HIV are recorded.

## References

[1] UNAIDS AIDSinfo [homepage on the internet] 2017 [cited 2017 July 20]. Available from http://www.unaids.org/en/resources/documents/2017/20170720/_global_aids_update_2017

[2] World Health Organization (WHO). Gender inequalities and HIV [homepage on the internet] 2013 [cited 2013 Feb 23] Available from http://www.who.int/gender/hiv_aids/en/

[3] Wathula J. Gender inequality dynamics in the prevention of a heterosexual HIV epidemic in sub-Saharan Africa. Afr J AIDS Res (AJAR). 2016;15(1):55–66. https://doi.org/10.2989/16085906.2016.1150310 27002358 10.2989/16085906.2016.1150310

[4] SANAC National Strategic Plan for HIV, TB and STIs 2017-–2022 [homepage on the internet]. 2017 [cited 2017 Mar 31]. Available from http://sanac.org.za/wp-content/uploads/2017/05/nsp_fulldocument_final.pdf

[5] United States Agency for International Development (USAID) United States strategy to prevent and respond to gender-based violence globally. Washington: USAID; 2013.

[6] Kaler A, Watkins SC, Angoti N Making meaning in the time of AIDS: Longitudinal narratives from the Malawi Journals Project. Afr J AIDS Res (AJAR). 2015;14(4):303–314. https://doi.org/10.2989/16085906.2015.1084342

[7] Tong R Feminist thought: A more comprehensive introduction. New York: Westview Press; 2009.

[8] Klaas NE, Peu MD, Netangaheni TR The perceptions of the farming community on HIV and AIDS in the Limpopo Province. Asian J Agriculture Rural Dev (JARD). 2014;4(8):437–448.

[9] Shisana O, Rehle T, Simbayi LC, et al South African National HIV Prevalence, Incidence and Behavior Survey. Cape Town: HSRC Press; 2014.

[10] Soko M, Moyo S, Rusinga O, Zhoushe A Risk factors of HIV infection among farm workers at Rattray Arnold Research Farm in Goromonzi district, Zimbabwe: A qualitative study. Afr J AIDS Res (AJAR). 2015;14(4):343–351. https://doi.org/10.2989/16085906.2015.1117003

[11] Polit FDL, Beck CT Nursing research: Generating and assessing evidence for nursing practice. 10th ed. Philadelphia, PA: Lippincott Williams & Wilkins; 2012.

[12] De Vos AS, Strydom H, Fouché CB, Delport CLS Research at grass roots: For the social sciences and human service professions. 4th ed. Cape Town: Creda Communications; 2011, p. 359.

[13] Liamputtong P Qualitative research methods. 3rd ed. South Melbourne: Oxford University Press; 2011, p. 278.

[14] Burns N, Grove SK Understanding nursing research, building evidence based practice. 5th ed. Philadelphia, PA: Elsevier Saunders; 2011, p. 585.

[15] Statistics South Africa Community Survey 2016 [cited 2016 July 01]. Available from: www.statssa.gov.za/?page_id=6283

[16] Leclerc-Madlala S, Simbayi LC, Cloete A The sociocultural aspects of HIV/AIDS in South Africa In: Rohleder P, Swartz L, Kalichman SC, Simbayi LC, editors HIV/AIDS in South Africa 25 years on: Psychosocial perspectives. New York: Springer; 2009; p. 13–26.

[17] Peacock D South Africa’s Sonke Gender Justice Network: Educating men for gender equality. Agenda. 2013;27(1):128–140. https://doi.org/10.1080/10130950.2013.808793

[18] Jewkes R, Dunkle KL, Nduna M, Shai N Intimate partner violence, relationship power inequity, and incidence of HIV infection in young women in South Africa: A cohort study. Lancet. https://doi.org/10.1016/S0140-6736(10)60548-X 10.1016/S0140-6736(10)60548-X 20557928

[19] Moussavi S, Amin A, Tobias A, et al Addressing gender inequality in HIV: A framework for gender-sensitive monitoring & evaluation. Journal of the International AIDS Society. 2015;18(Suppl 5):20302. 26643464 10.7448/IAS.18.6.20302 PMC4672401

[20] Shisana O, Rehle T, Simbayi LC, et al South African national HIV prevalence, incidence, behaviour and communication survey 2008: A turning tide among teenagers. Cape Town: HSRC Press; 2009.

